# Regulatory T-Cell Enhancement, Expression of Adhesion Molecules, and Production of Anti-Inflammatory Factors Are Differentially Modulated by Spheroid-Cultured Mesenchymal Stem Cells

**DOI:** 10.3390/ijms232214349

**Published:** 2022-11-18

**Authors:** Amandda Évelin Silva-Carvalho, Ingrid Gracielle Martins da Silva, José Raimundo Corrêa, Felipe Saldanha-Araujo

**Affiliations:** 1 Hematology and Stem Cells Laboratory, University of Brasília, Brasilia 70910-900, Brazil; 2 Molecular Pharmacology Laboratory, University of Brasília, Brasilia 70910-900, Brazil; 3 Microscopy and Microanalysis Laboratory, University of Brasília, Brasilia 70910-900, Brazil

**Keywords:** mesenchymal stem cells, three-dimensional spheroids, T-cells, adhesion molecules, ICAM-1

## Abstract

The culture of mesenchymal stem cells (MSCs) as spheroids promotes a more physiological cellular behavior, as it more accurately reflects the biological microenvironment. Nevertheless, mixed results have been found regarding the immunosuppressive properties of spheroid-cultured MSCs (3D-MSCs), the mechanisms of immunoregulation of 3D-MSCs being scarcely described at this point. In the present study, we constructed spheroids from MSCs and compared their immunosuppressive potential with that of MSCs cultured in monolayer (2D-MSCs). First, we evaluated the ability of 2D-MSCs and 3D-MSCs to control the activation and proliferation of T-cells. Next, we evaluated the percentage of regulatory T-cells (Tregs) after the co-culturing of peripheral blood mononuclear cells (PBMCs) with 2D-MSCs and 3D-MSCs. Finally, we investigated the expression of adhesion molecules, as well as the expressions of several anti-inflammatory transcripts in 2D-MSCs and 3D-MSCs maintained in both inflammatory and non-inflammatory conditions. Interestingly, our data show that several anti-inflammatory genes are up-regulated in 3D-MSCs, and that these cells can control T-cell proliferation. Nevertheless, 2D-MSCs are more efficient in suppressing the immune cell proliferation. Importantly, contrary to what was observed in 3D-MSCs, the expressions of ICAM-1 and VCAM-1 are significantly upregulated in 2D-MSCs exposed to an inflammatory environment. Furthermore, only 2D-MSCs are able to promote the enhancement of Tregs. Taken together, our data clearly show that the immunosuppressive potential of MSCs is significantly impacted by their shape, and highlights the important role of cell–cell adhesion molecules for optimal MSC immunomodulatory function.

## 1. Introduction

MSCs are multipotent progenitor cells that can be harvested from various adult tissues [1] and easily expanded in vitro. Such cells possess several biological characteristics which make them attractive tools in the fields of regenerative medicine and cellular therapy. In response to stimuli mediated by cytokines and other biomolecules, MSCs can migrate to injured tissues and produce and secrete several factors that regulate signaling pathways associated with inflammatory responses, tissue repair, angiogenesis, apoptosis, and antimicrobial control [2,3].

Although the mechanisms of immunomodulation are not completely understood, they seem to involve cell–cell contact, the secretion of soluble factors, and the generation of classical and non-classical Tregs [4,5]. Importantly, MSCs’ immunosuppressive potential has been explored to treat immune- and inflammatory-related diseases, such as systemic lupus erythematosus [6] and Graft-versus-host disease (GVHD) [7]. Currently, clinical trials are also evaluating the effectiveness of MSCs in treating patients with COVID-19 [8,9,10]. However, MSCs therapy still faces several challenges, including the high number of cells required for therapy and the lack of standardization in cultivation methods, doses, and routes of administration [11]. Differences between in vitro and in vivo results have been attributed to the fact that the traditional model of cell culture—in which cells remain attached to a plastic surface—is not able to promote a similar environment compared with the one experienced by cells in vivo [12,13,14]. Such a different condition has been related to the heterogeneous responses observed in clinical trials, highlighting the urgent need for further optimization of MSC-based therapy. In this line, several strategies have been adopted to optimize the immunomodulatory capacity of MSCs, including pre-conditioning with pro-inflammatory factors or hypoxia and the three-dimensional culture model of such cells [15].

In vivo studies have shown that MSCs tend to form small aggregates after infusion [16,17,18], and that this conformation can dramatically modify their biological properties [19,20]. Indeed, most of the data regarding MSCs’ immunosuppressive potential obtained so far is based on the monolayer adherent culture method, in which cells are kept in contact with plastic adherent surfaces. Meanwhile, assembly to aggregates appears to activate MSCs, increasing their self-renewal, as well as regenerative [21] and immunoregulatory, activities [22,23], although the fundamental mechanism underlying these effects is not well understood. Other authors have even pointed out that the formation of 3D-MSCs impairs their immunosuppressive potential [24], justifying more detailed investigations regarding the impact of three-dimensional culture in different aspects of MSC immunosuppression. Here, we produced 3D-MSCs obtained from adipose tissue and compared their immunosuppressive potential with the cells maintained in the monolayer. Next, we explored the immunomodulation mechanisms of 2D-MSCs and 3D-MSCs, characterizing the expression of adhesion molecules on their surface, the enhancement of classical and non-classical Tregs by these cells, and their expression of anti-inflammatory factors.

## 2. Results

### 2.1. Spheroid Formation and Morphological Analysis

**Figure 1:**
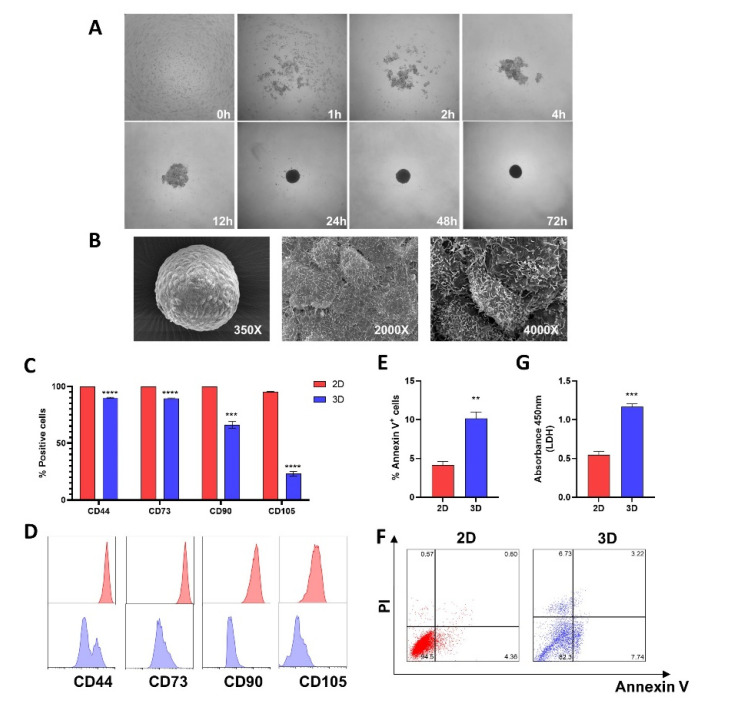
Spheroid formation and MSCs characterization. (**A**) Chronological record of 3D-MSCs compaction. Images were captured at 0, 1, 2, 4, 12, 24, 48, and 72 h after 3D-MSCs seeding on 96-well agarose-coated plates at 4 × magnification. (**B**) Scanning electron microscopy images. From left to right the magnifications are 350, 2000, and 4000 ×. (**C**) CD44, CD73, CD90, and CD105 expression on 2D-MSCs (2D) and 3D-MSCs (3D). (**D**) Representative histograms of one MSC sample evaluated. Red color corresponds with 2D-MSCs, while blue color corresponds with 3D-MSCs. (**E**) Percentages of apoptotic (annexin V+) 2D and 3D-MSCs evaluated after their dissociation with collagenase IA. (**F**) Representative flow cytometric dot plots showing expressions of PI and Annexin V in 2D and 3D-MSCs. (**G**) LDH release by 2D and 3D-MSCs. ** *p* < 0.01; *** *p* < 0.001; **** *p* < 0.0001.

To generate 3D-MSCs, the cells were dissociated from the monolayer culture and plated in 96-well agarose-coated plates. After an incubation period of 3 days, single and homogenous 3D-MSCs were spontaneously generated (Figure 1A). In the first 48 h, all of the cells were already incorporated into spheroids. However, between 48–72 h, the spheroids became more compacted. Scanning microscopy analysis was used to confirm the regular spheroid shape and to determine the mean spheroid diameter after 72 h, which was approximately 254 µm (Figure 1B).

### 2.2. MSCs Characterization

Compared with 2D-MSCs, 3D-MSCs presented a significant reduction in the expression of CD44 (*p* < 0.0001), CD73 (*p* < 0.0001), CD90 (*p* < 0.001), and CD105 (*p* < 0.0001) (Figure 1C,D).

### 2.3. Assembly of MSCs into 3D-MSCs Increases Apoptosis and LDH Release

Due to the fact that the spheroid size can affect the oxygen availability to cells on the core, we compared the impact of different culture models on MSC viability. After 72 h, an increase of Annexin-V-positive cells was detected in 3D-MSCs in contrast with 2D-MSCs samples (*p* = 0.001) (Figure 1E,F). To validate such findings, we also collected the supernatant from both cultures to measure their LDH releases. Accordingly, there was an increase in LDH release from 3D-MSCs cultures (*p* = 0.0002) (Figure 1G).

### 2.4. 2D-MSCs Exhibit a Higher Immunosuppressive Potential Than 3D-MSCs

**Figure 2:**
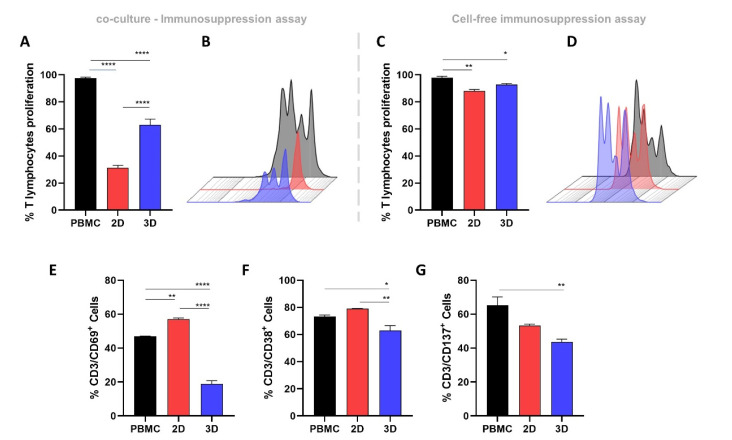
Determination of T-cell proliferation and activation. (**A**) The 2D-MSCs and 3D-MSCs inhibited T-cell proliferation in 72 h of co-culture. (**B**) Representative CFSE histograms of PHA-activated PBMCs (black), PHA-activated PBMCs cultured with 2D-MSCs (red), and PHA-activated PBMCs cultured with 3D-MSCs (blue). (**C**) The 2D-MSCs’ and 3D-MSCs’ secretomes inhibited T-cell proliferation in 72 h of culture. (**D**) Representative CFSE histograms of PHA-activated PBMCs (black), PHA-activated PBMCs cultured with 2D-MSCs secretome (red), and PHA-activated PBMCs cultured with 3D-MSCs secretome (blue). (**E**) The 2D-MSCs promoted and 3D-MSCs inhibited CD69 expression on T-cells in 24 h of co-culture. (**F**,**G**) The 3D-MSCs inhibited CD38 and CD137 expression on T-cells in 24 h of co-culture. * *p* < 0.05; ** *p* < 0.01; **** *p* < 0.0001.

Both 2D- and 3D-MSCs were able to suppress T-cell proliferation (*p* < 0.0001). However, 2D-MSCs exhibited a higher immunosuppressive potential when compared with 3D-MSCs (*p* < 0.0001) (Figure 2A,B). To verify the immunoregulatory potentials of the 2D-MSCs’ and 3D-MSCs’ secretomes, we also evaluated the effects of their conditioned mediums on T-cell proliferation. Although the suppressive effect was lower when compared with the co-culture assay, the 2D-MSCs conditioned medium (*p* = 0.001) still presented a more suppressive capacity than the conditioned medium obtained from 3D-MSCs (*p* = 0.04) (Figure 2C,D).

### 2.5. 3D-MSCs Promote a Superior Suppression of T-Cell Activation Markers Compared with 2D-MSCs

To verify the impact of the MSC culture models on their capacity to reduce T-cell activation, we evaluated the expression of CD38, CD69, and CD137 on PHA-activated PBMCs after 24 h of co-culture with 2D- and 3D-MSCs. Interestingly, only the 3D-cultured MSCs were able to significantly reduce the expression of all T-cell markers CD38 (*p* = 0.03), CD69 (*p* < 0.0001), and CD137 (*p* = 0.006). On the other hand, we observed an increase in CD69 expression on the PBMCs co-cultured with 2D-MSCs (*p* = 0.002) (Figure 2E–G).

### 2.6. 2D-MSCs Induce the Enhancement of Classical Treg Cells, while 3D-MSCs Reduce the Levels of Non-Classical CD8^+^CD28^−^ Tregs

**Figure 3:**
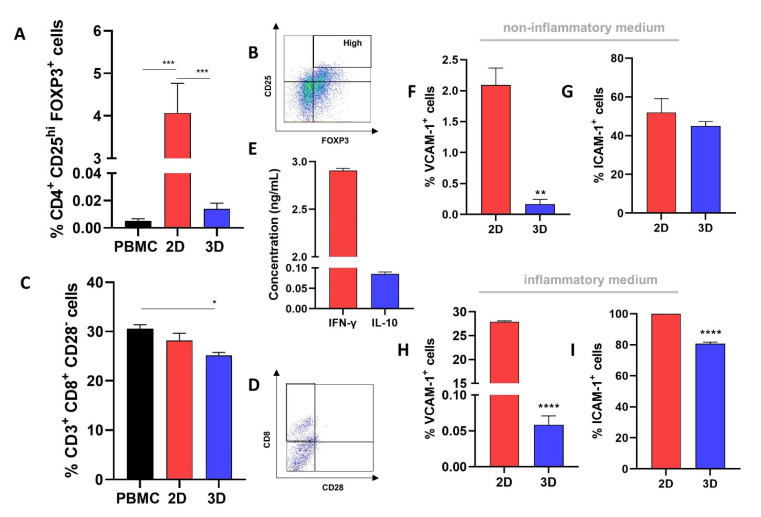
Treg and adhesion molecule induction by 2D-MSCs and 3D-MSCs. (**A**) Enhancement of CD4^+^ CD25^high^ FOXP3^+^ Tregs cells by 2D-MSCs. (**B**) Gating strategy for analysis of CD25^hi^FOXP3^+^ population in CD4 cells. (**C**) Reduction of CD8^+^CD28^−^ Treg cells in PBMCs co-cultured with 3D-MSCs. (**D**) Gating strategy for analysis of CD8^+^CD28^−^ population in CD3^+^ cells. (**E**) Levels of IFN-γ and IL-10 in the inflammatory medium produced by PHA-activated PBMCs. (**F**,**G**) VCAM-1 and ICAM-1 expression on 2D-MSCs and 3D-MSCs cultured in non-inflammatory conditions. (**H**,**I**) VCAM-1 and ICAM-1 expression on 2D-MSCs and 3D-MSCs cultured in inflammatory medium. * *p* < 0.05; ** *p* < 0.01; *** *p* < 0.001, **** *p* < 0.0001.

Treg generation is an important mechanism of immunosuppression by MSCs [25]. Our data shows that 2D-MSCs are able to promote a significant enhancement of the classical CD4^+^ CD25^high^ FOXP3^+^ Tregs population compared with the PHA-activated PBMC (*p* = 0.0009) and 3D-MSCs (*p* = 0.0009) (Figure 3A,B). In parallel, we observed a slight decrease in the population of non-classical CD8^+^CD28^-^ Treg cells in PBMCs co-cultured with 3D-MSCs (*p* = 0.02) (Figure 3C,D).

### 2.7. ICAM-1 and VCAM-1 Expression Is Higher in 2D-MSCs Exposed to an Inflammatory Medium

The inflammatory media produced by PHA-activated PBMCs was characterized and used for experiments investigating the effects of inflammatory conditioned media on 2D- and 3D-MSC functions. The conditioned media presented 2.90 ng/mL of INF-γ and 0.08 ng/mL of IL-10 (average) (Figure 3E).

Considering the role of ICAM-1 and VCAM-1 in immunosuppression mediated by MSCs, we determined the ICAM-1 and VCAM-1 levels in 2D- and 3D-MSCs when these cells were exposed or not to inflammation. We observed that 2D-MSCs presented a higher expression of VCAM-1 than 3D-MSCs (*p* = 0.001) in a non-inflammatory context (Figure 3F). No differences were observed in ICAM-1 expressions between the samples (Figure 3G). However, when exposed to the inflammatory medium, there was a significant increase in both ICAM-1 (*p* < 0.0001) and VCAM-1 (*p* < 0.0001) expressions on the 2D-MSCs compared with the 3D-MSCs (Figure 3H,I).

### 2.8. MSC Culture Condition Impacts the mRNA Expressions of Different Immune-Related Genes

**Figure 4:**
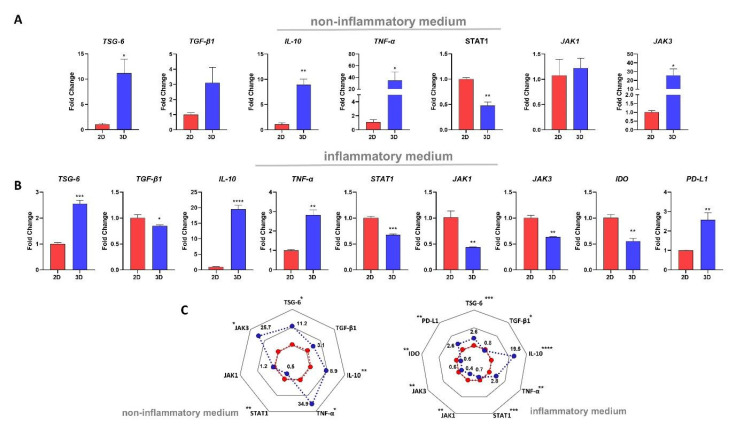
Transcriptional profile of 2D-MSCs and 3D-MSCs cultured in inflammatory or non-inflammatory conditions. (**A**) TSG-6, TGF-β1, IL-10, TNF-α, STAT1, JAK1, and JAK3 expressions in 2D-MSCs and 3D-MSCs exposed to a non-inflammatory medium. Cells did not express IDO and PD-L1 when in a non-inflammatory medium. (**B**) TSG-6, TGF-β1, IL-10, TNF-α, STAT1, JAK1, JAK3, IDO, and PD-L1 expressions in 2D-MSCs and 3D-MSCs exposed to an inflammatory medium. The median Ct values obtained 2D-MSCs were used as a reference. (**C**) Radial plot demonstrating the differences in overall transcripts between 2D-MSCs and 3D-MSCs cultured in inflammatory or non-inflammatory conditions. * *p* < 0.05; ** *p* < 0.01; *** *p* < 0.001; **** *p* < 0.0001.

We noticed different patterns in 2D- and 3D-MSCs transcriptional profiles when they were exposed or not to inflammation. In non-inflammatory conditions, 3D-MSCs exhibited a higher expression of TSG-6 (*p* = 0.01), IL-10 (*p* = 0.001), TNF-α (*p* = 0.04), and JAK3 (*p* = 0.01) and a decrease in STAT1 (*p* = 0.001) (Figure 4A) when compared with 2D-MSCs. IDO and PD-L1 expressions were not detectable in either group. However, when these cells were cultured in an inflammatory medium, the fold differences between TSG-6 (*p* = 0.0002) and TNF-α (*p* = 0.001) between 2D- and 3D-MSCs were lower. Although there was an increase in IL-10 expression (*p* < 0.0001) and PD-L1 (*p* = 0.006), there was a downregulation of TGF-β1 (*p* = 0.03), IDO (0.002), STAT1 (*p* = 0.006), JAK1 (*p* = 0.004), and JAK3 (*p* = 0.01) in 3D-MSCs (Figure 4B).

## 3. Discussion

In this study, we investigated the immunosuppressive effects of 2D- and 3D-MSCs on T-cells, and demonstrated that the cell culture condition of MSCs significantly impacts the production of anti-inflammatory factors, the enhancement of Tregs, and the expression of cell–cell adhesion molecules. Initially, we aimed to establish a reproducible method for generating MSC spheroids. Using a previously described protocol [26], we observed that after 72 h, MSCs were able to spontaneously aggregate into size and morphological homogenous spheroids. Each spheroid was formed by 25,000 cells, to mimic the formation of the spontaneous aggregates that occurs after an injection of a high-density cell suspension [18]. The characterization by flow cytometry revealed that 3D-MSCs present a decreased expression of several MSC markers defined by ISCT [27], such as CD44, CD90, and CD105, compared with 2D-MSCs, which were similarly reported by others [23,28,29]. Interestingly, it was suggested that the reduction of such markers may reflect a shift between the need for MSCs to adhere to plastic surfaces in monolayer cultures and the new environment found in the 3D model, where the contact of these cells is exclusively maintained with other cells and with extracellular matrix components [30]. In this line, it was reported that CD105 seems to have an important role in spheroid compaction, given that only CD105^+^ synovial membrane MSCs were able to form spheroids [29].

The size of spheroids also impacts the oxygen diffusion to the cells located in the core. It was reported that cellular aggregates bigger than 200 µm can suffer from severe oxygen limitations [31,32]. However, in a study conducted by Murphy and colleagues, no hypoxic core was detected in MSCs spheroids composed of up to 60,000 cells [33]. In agreement with previous reports [23,34], we found slightly higher levels of apoptosis in 3D-MSCs cells compared with 2D-MSCs. We then analyzed the viability of MSCs, and detected more cells under apoptosis in 3D-MSCs compared with 2D-MSCs. It is unclear whether apoptosis compromises the function of spheroid-cultured MSCs, due to the fact that the assembly of MSCs into spheres appears to trigger caspase-dependent IL-1 signaling and stimulates the secretion of various modulators of inflammation and immunity [18].

Importantly, the assembly of MSCs into spheroids has been explored as a strategy to optimize their immunosuppressive paracrine effect. Several lines of evidence suggest that MSC aggregates possess a different transcriptional profile characterized by the up-regulation of several immunomodulatory transcripts, such as TSG-6 [23], IL-10 [35], and PGE2 [18]. In vitro studies also showed that spheroid-conditioned media can polarize macrophages from an M1 pro-inflammatory phenotype to an anti-inflammatory M2 phenotype [22,36]. In addition, in the spheroid shape, MSCs were shown to control T lymphocyte proliferation more efficiently than their 2D-cultured counterparts [37]. Despite these results, compromised immunosuppressive capacity by 3D-MSCs was also reported [24]. In our hands, despite 3D-MSCs having higher transcriptional levels of anti-inflammatory factors, including TSG-6 and IL-10, 2D-MSCs exhibited a superior suppressive capacity to control T-cell proliferation. Such findings are particularly interesting, especially because 3D-MSCs have been proposed as an alternative to boost MSCs’ immunosuppressive properties [15,38]. To better understand the differences found, we cultured 2D- and 3D-MSCs in an inflammatory medium generated by PHA-activated PBMCs, and observed that, when exposed to inflammation, 3D-MSCs present a reduction in the expression of IDO and TGF-β1, compared with their 2D-cultured counterparts. Importantly, IDO is one of the major mechanisms used by human MSCs to suppress T-cell proliferation [39,40], and it also controls TSG-6-mediated anti-inflammatory therapeutic effects [41]. Its production is drastically increased in IFN-γ-activated MSCs as a result of STAT1 signaling [42,43]. Janus kinase and signal transducers and activators of transcription (JAK-STAT) are critical mediators of cytokine’s and growth factor’s responses [44], and seem to be involved in the regulation of human MSCs immunosuppressive properties [42,45]. Enhanced STAT1 phosphorylation was also observed after MSCs interacted with activated PBMCs secretome, and this effect was completely blocked after Ruxolitinib treatment, a JAK1/JAK2 inhibitor [45]. Accordingly, the low levels of IDO on 3D-MSCs were followed by a reduction in STAT1, JAK1, and JAK3 expressions. These data indicate that the gene-expression pattern of 3D-MSCs changes during inflammation, leading to a reduction of some crucial molecules involved in MSCs immunosuppression.

In addition to controlling the division of T-cells, in the first 24 h of co-culture with PBMCs, 3D-MSCs reduced the expression of the activation markers CD38, CD69, and CD137 on T-cells. In contrast, 2D-MSCs induced an increased expression of CD69 on T-cells. Interestingly, there are conflicting results in the literature about the effects of 2D-MSCs on CD69 expression in T-cells [46,47,48]. In fact, it has been demonstrated that the immunosuppressive behavior of MSCs is dynamic, and that before controlling T-cell division, MSCs can induce a transient increase in the activation of such cells, which is accompanied by the secretion of IL- 2 and INF-γ [49]. Using MSCs from bone marrow, our group demonstrated that by activating the non-canonical NF-kB pathway, MSCs can sustain CD69 expression on T-cells as an immunosuppressive marker [50].

Considering that contact between MSCs and T-cells is a crucial mechanism for immunoregulation [51], we investigated the expression of the immunosuppressive molecules VCAM-1 and ICAM-1 on 2D- and 3D-MSCs after exposing the cells to a non-inflammatory or an inflammatory environment. We observed that 3D-MSCs presented a lower level of VCAM-1 compared with 2D-MSCs. Interestingly, when exposed to the inflammatory environment, both MSCs showed an increase in ICAM-1 expression, and in 3D-MSCs this increase was significantly more pronounced. Furthermore, only 2D-MSCs showed increased VCAM-1 expression on their surface when challenged by an inflammatory environment. These data show that the shape of MSCs significantly impacts the expression of adhesion molecules, and that the higher expression of such molecules in 2D-MSCs is in line with the differential potential of these cells and 3D-MSCs to control T-cell proliferation. Possibly, the physical interaction between 3D-MSCs with PBMCs is limited, as only MSCs that are on the external face of the spheroid are able to exert cell–cell contact with T-cells, justifying our observations regarding the 3D-MSC expression of adhesion molecules and the lower immunosuppressive potential in co-culture assays. Another point that reinforced the importance of adhesion molecules in the immune control exerted by MSCs was the marked decrease in the immunosuppressive potential of 2D- and 3D-MSCs noticed when PBMCs were only exposed to the supernatants of these cells.

Although the mechanisms of Treg generation by MSCs are not completely understood, the expansion of Treg seems to be dependent on cell-to-cell interactions between MSCs and T-cells [52]. Here, we investigated whether the greater levels of adhesion molecules and the highest suppressive effect observed in 2D-MSCs could be associated with Treg enhancement. In fact, 2D-MSCs were able to induce an increased number of the classical CD4^+^ CD25^high^FOXP3^+^ Treg cells compared with 3D-MSCs or PHA-activated PBMCs cultured alone. Importantly, it was demonstrated in a GVHD mice model that the infusion of MSCs overexpressing ICAM-1 increases the immunosuppressive effect on T- cells and promotes Tregs generation [53]. Furthermore, it’s important to highlight that MSCs-derived factors such as TGF-β1 [54] and IDO [55] are also involved in Treg induction, and both were downregulated in 3D-MSCs exposed to inflammation.

In conclusion, our data shows that even though the expression of several anti-inflammatory genes is upregulated in 3D-MSCs, it is not sufficient to maximize their immunosuppressive function. In contrast, 2D-MSCs exhibit a superior immunosuppressive potential, possibly due to the enhanced cell–cell adhesion molecule expression. These results clearly show that the immunosuppressive potential of MSCs is significantly impacted by their shape, and contribute to a better understanding of the interactions that occur between MSCs and T-cells.

## 4. Materials and Methods

### 4.1. Adipose-Derived MSCs Culture

MSCs (n = 3) were obtained from three female healthy donors following a lipoaspiration procedure performed at the Clínica Carpaneda Cirurgia Plástica, Brazil. The cells were cultured in T75 flasks using alpha-minimum essential medium (α-MEM) supplemented with 15% fetal bovine serum (FBS—GIBCO, Waltham, MA, USA), 2 mM glutamine, and 100 U/mL penicillin/streptomycin (Sigma-Aldrich, St. Louis, MO, USA) at 37 °C and 5% CO_2_. The medium was changed every 2 days, and the cells were split when they reached 80–90% confluence. Cells were then dissociated with 0.05% Trypsin (GIBCO, Waltham, MA, USA), counted, and re-plated for monolayer expansion cultures. The study protocols were approved by the Institutional Ethics Committee, and written informed consent was obtained from all participants.

### 4.2. Spheroid Generation and Dissociation

For spheroid generation, MSCs were dissociated from culture flasks, suspended in a culture medium, and then seeded in 96-well plates coated with 50 μL of 1.5% agarose prepared in culture media (wt/vol) at a density of 25,000 cells per well [26]. The plates were incubated for 72 h in a humidified atmosphere with 5% CO_2_ at 37 °C for spheroid formation. The entire process was monitored using the Primo Vert inverted microscope (Zeiss, Jena, Germany). After the incubation period, the spheroids were collected from the plates and used for experimental assays. For dissociation, the spheroids were washed three times with PBS and incubated with 2 mg/mL of collagenase IA [56].

### 4.3. Scanning Electron Microscopy (SEM)

Spheroids were harvested from the plates, washed with PBS, and fixed with osmium tetroxide 1% (wt/vol) for 30 min. The samples were further dehydrated with increasing acetone concentrations (50–70–90–100%). Finally, the samples were critical-point dried and coated with gold to improve visualization. The diameter and morphology were confirmed using the MEV Jeol JSM-7001F scanning microscope.

### 4.4. Immunophenotypic Characterization

2D-MSCs and 3D-MSCs were phenotypically characterized according to the minimal criteria proposed by the International Society for Cellular Therapy (ISCT) using the BD Stemflow hMSCs Analysis kit, following the manufacturer’s instructions (Pharmingen, BD Biosciences, East Rutherford, NJ, USA). Ten thousand events were recorded for each sample, and data were acquired using a FACSCalibur flow cytometer. FlowJo software 10.0.7 (Ashland, OR, USA) was used for data analysis.

### 4.5. Viability Assay

2D- and 3D-MSC viabilities were determined by annexin V/PI staining using flow cytometry. Briefly, the cells were dissociated from the plates or the spheroids and stained with annexin V- FITC and PI, following the manufacturer’s instructions. Ten thousand events were recorded from each sample using a FACSCalibur flow cytometer. Early apoptotic (annexin^+^/PI^−^) and dead-cell (annexin^+^/PI^+^) populations were quantified using the FlowJo software 10.0.7 (FlowJo Llc., Ashland, OR, USA).

### 4.6. Determination of Lactate Dehydrogenase (LDH) Release

Supernatants from 2D-MSCs and 3D-MSCs cultures were collected and the LDH release measurement was performed using the CytoTox 96 Non-Radioactive Cytotoxicity Assay kit, according to the manufacturer’s instructions (Promega Corp., Madison, WI, USA). The absorbance was determined using a DTX 800 Series Multimode Detector (Beckman Coulter, Brea, CA, USA) at 490 nm.

### 4.7. Immunosuppression Assay

For the immunosuppression assay, PBMCs were obtained from healthy volunteers using Histopaque 1077 (Sigma-Aldrich, USA). After washing and counting, the cells were stained with 2.5 μM carboxyfluorescein succinimidyl ester (CFSE) to allow for proliferation analysis. The labeled PBMCs were then activated with 5 μg/mL of phytohaemagglutinin (PHA, Sigma-Aldrich, St. Louis, MO, USA) and co-cultured with 2D- and 3D-MSCs at a 10:1 ratio for 72 h. To evaluate the immunosuppressive effects of 2D- and 3D-MSCs secretome, their conditioned mediums were previously collected and added to PHA-activated CFSE-labeled PBMCs. After 72 h, PBMCs from both conditions were recovered, stained with anti-CD3 APC (Invitrogen, Waltham, MA, USA), and the proliferation of T-cells was determined by flow cytometry after the collection of ten thousand events. The analysis was performed using FlowJo software 10.0.7 (FlowJo Llc., Ashland, OR, USA).

### 4.8. T-Cell Activation Assay

To evaluate the impact of 2D- and 3D-MSCs on T-cell activation, these cells were co-cultured with PHA-activated PBMCs for 24 h in the same ratio as used in the immunosuppression assay. After this, cells were collected, washed, and stained with anti-CD3 PerCP, anti-CD38 APC, anti-CD69 FITC, and anti-CD137 PE. All antibody used were acquired from Invitrogen (Waltham, MA, USA). Ten thousand events were collected and analyzed by flow cytometry.

### 4.9. Treg Quantification Assay

To determine Treg generations by 2D- and 3D-MSCs, the cells were co-cultured with PHA-activated PBMCs at a 1:10 ratio for 72 h. Then, PBMCs were recovered and stained with anti-CD4 FITC, anti-CD25 APC, and FOXP3 PE, according to the manufacturer’s recommendations (FoxP3 Staining Kit, BD Biosciences). One hundred thousand events were recorded for each sample in a FACSCalibur flow cytometer. Additionally, the cells were stained with anti-CD3 PerCP, anti-CD8 PE (Invitrogen, Waltham, MA, USA), and anti-CD28 FITC (Invitrogen, Waltham, MA, USA) for non-classical CD8^+^CD28^−^ Treg quantification [57]. For this, twenty thousand events were recorded. Analyses were performed using FlowJo software 10.0.7 (FlowJo Llc., Ashland, OR, USA).

### 4.10. Inflammatory Medium Obtention

Due to the fact that exposure to inflammation can modulate MSCs suppressive capacity [58], we collected the supernatant of PHA-activated PBMCs to mimic an inflammatory environment and evaluate the response of 2D-MSCs and 3D-MSCs in this milieu. For this, 1 × 10^6^ PBMCs were seeded in 24 wells plates containing RPMI 1640 medium (GIBCO) supplemented with 15% FBS. PBMCs were activated with 5 µg/mL of PHA for 72 h. The medium was then collected, centrifuged to remove cell debris, and used for the following assays.

### 4.11. Enzyme-Linked Immunosorbent Assay

The inflammatory medium generated by PHA-activated PBMCs was characterized according to the concentrations of IFN-γ and IL-10 by ELISA, following the manufacturer’s instructions (ImmunoTools, Friesoythe, Germany). The absorbance was measured at 450 nm using the automatic microplate reader DTX 800 Multimode Detector (Beckman Coulter), and the concentration was determined using standard curve values.

### 4.12. VCAM-1 and ICAM-1 Expression

Considering the crucial role of ICAM-1 and VCAM-1 in the immunoregulatory mechanisms of MSCs, we analyzed the expressions of these proteins in 2D- and 3D-MSCs exposed or not to an inflammatory milieu by flow cytometry. Briefly, 2D- and 3D-MSCs were cultured for 72 h in RPMI (non-inflammatory) or an inflammatory medium, and after this period the cells were recovered and stained with anti-CD54 APC (BD Biosciences) and anti-CD106 FITC (BD Biosciences). Ten thousand events were recorded, and analyses were performed using FlowJo software 10.0.7 (FlowJo Llc., Ashland, OR, USA).

### 4.13. Real-Time PCR

Gene expression analyses were performed in 2D- and 3D-MSCs exposed to both fresh RPMI and conditioned media derived from activated (inflammatory) or resting PBMCs (non-inflammatory medium). RNA extraction was performed using the TRI reagent, according to the manufacturer’s recommendation (Sigma-Aldrich). The amounts and qualities of the samples were determined using Nanodrop one (Thermo Scientific, Waltham, MA, USA). The total RNA was converted to single-stranded cDNA using the High-Capacity cDNA Reverse Transcription Kit (Applied BioSystems, Waltham, MA, USA). Quantitative PCR was performed using TaqMan probes (ThermoFisher, Waltham, MA, USA) for TGF-β amplification (Hs00248373_m1). Transcriptional levels of TSG-6, IL-10, TNF-α, JAK1, JAK3, STAT1, IDO, and PD-L1 were investigated using GoTaq qPCR Master Mix (Promega Corp., USA), following the manufacturer’s instructions. The sequence of the primers used is listed in Supplementary Table S1. The reactions were performed in technical duplicates, and the relative fold change was obtained by the 2^−ΔΔCt^ method [59]. The median Ct values obtained from the samples of 2D-MSCs were used as a reference.

### 4.14. Statistical Analysis

Data were reported as mean ± SEM, and all analyses were performed using Prism 9 software (GraphPad Software Inc., San Diego, CA, USA). The statistical significance was calculated using Student’s *t*-test analyses to compare the differences between two groups and using ANOVA to compare three or more experimental groups. The value of *p* < 0.05 was considered statistically significant.

## Data Availability

All data to support the findings are fully available.

## Supplementary Materials

The following supporting information can be downloaded at: https://www.mdpi.com/article/10.3390/ijms232214349/s1.
