# Supplementary material for: Regulatory T-Cell Enhancement, Expression of Adhesion Molecules, and Production of Anti-Inflammatory Factors Are Differentially Modulated by Spheroid-Cultured Mesenchymal Stem Cells
Source: Int J Mol Sci. 2022 Nov 18;23(22):14349. doi: 10.3390/ijms232214349 (PMC9695986; doi:10.3390/ijms232214349)
Supplement: Supplementary file 1 [file ijms-23-14349-s001.zip › ijms-1991839-supplementary.pdf]

Table S1. Primer sequences.

Gene	Primer	Sequence (5′- 3′)
GAPDH	Foward	TCAACGACCACTTTGTCAAGCTCAGCT
	Reverse	GGTGGTCCAGGGGTCTTAC
TSG-6	Foward	CCCAGGTTGCTTGGCTGATT
	Reverse	GGACCCATACGTACCTTCCC
IL-10	Foward	GGCACCCAGTCTGAGAACAG
	Reverse	ACTCTGCTGAAGGCATCTCG
TNF-α	Foward	CACAGTGAAGTGCTGGCAAC
	Reverse	GATCAAAGCTGTAGGCCCCA
JAK-1	Foward	CCACTACCGGATGAGGTTCTA
	Reverse	GGGTCTCGAATAGGAGCCAG
JAK-3	Foward	GCCTGGAGTGGCATGAGAA
	Reverse	CCCCGGTAAATCTTGGTGAA
STAT-1	Foward	GTTATGGGACCGCACCTTCA
	Reverse	CAGTGAACTGGACCCCTGTC
IDO	Foward	GGGAAGCTTATGACGCCTGT
	Reverse	CTGGCTTGCAGGAATCAGGA
PD-L1	Foward	AAACAATTAGACCTGGCTG
	Reverse	TCTTACCACTCAGGACTTG
